# Gluten Unraveled: Latest Insights on Terminology, Diagnosis, Pathophysiology, Dietary Strategies, and Intestinal Microbiota Modulations—A Decade in Review

**DOI:** 10.3390/nu16213636

**Published:** 2024-10-25

**Authors:** Dana Stanciu, Hristian Staykov, Stela Dragomanova, Lyubka Tancheva, Radu Samuel Pop, Irina Ielciu, Gianina Crișan

**Affiliations:** 1 Department of Pharmaceutical Botany, Faculty of Pharmacy, “Iuliu Hațieganu” University of Medicine and Pharmacy, 400337 Cluj-Napoca, Romania; dana.stanciu@elearn.umfcluj.ro (D.S.); irina.ielciu@umfcluj.ro (I.I.); gcrisan@umfcluj.ro (G.C.); 2 Department of Pharmacology and Toxicology, Faculty of Medicine, Medical University of Sofia, 1431 Sofia, Bulgaria; 3 Department of Pharmacology, Toxicology and Pharmacotherapy, Faculty of Pharmacy, Medical University of Varna, 9000 Varna, Bulgaria; stela_dragomanova@abv.bg; 4 Institute of Neurobiology, Bulgarian Academy of Sciences, 1113 Sofia, Bulgaria; lyubkatancheva@gmail.com; 5 3rd Department of Pediatrics, “Iuliu Hațieganu” University of Medicine and Pharmacy, 400217 Cluj-Napoca, Romania; radusamuelpop@gmail.com

**Keywords:** celiac disease, non-celiac gluten sensitivity, gluten-free diet, nutritional status, clinical nutrition, therapeutic interventions, micronutrient deficiencies, intestinal microbiota modulations, dietary management, dysbiosis

## Abstract

A decade of research on gluten-related disorders (GRDs) is reviewed in this study, with a particular emphasis on celiac disease (CD) and non-celiac gluten sensitivity (NCGS). GRDs are triggered by the ingestion of gluten and gluten-like proteins found in wheat, barley, and rye. These proteins lead to intestinal damage in celiac disease, an autoimmune condition characterized by villous atrophy and a variety of gastrointestinal and extraintestinal symptoms. More enigmatic and less understood, NCGS involves symptoms similar to CD but without the immunological reaction or intestinal damage. Recent years have seen advances in the understanding of GRDs, particularly in connection to how intestinal microbiota influences disease progression and patient outcomes. The gluten-free diet (GFD) is still the standard therapy recommended for GRDs despite significant challenges, as discussed in this article. Precise diagnostic methods, patient education and dietary counseling are critical for improving patients’ quality of life. The purpose of this review is to provide a more clear and up-to-date understanding of GRDs, and to help further research on this important topic.

## 1. Introduction

Numerous disorders, such as celiac disease (CD), dermatitis herpetiformis, gluten ataxia, peripheral neuropathy, wheat allergy (WA), irritable bowel syndrome (IBS), and even a “newer” syndrome known as “non-celiac gluten sensitivity (NCGS)” have been associated with gluten consumption [1,2,3]. “Gluten-related disorders (GRDs)” is an umbrella term for a number of immune-mediated conditions related to gluten ingestion, mainly CD, NCGS, and WA [4], and in this article, it is used to refer to the first two.

There is a wide range of clinical symptoms associated with GRDs, including both extraintestinal and gastrointestinal manifestations, impacting nearly every organ in the human body after gluten exposure in predisposed individuals [5]. The purpose of this review is to provide a clear and up-to-date understanding of CD and NCGS by examining their similarities and differences, definitions, pathophysiology, clinical signs and symptoms, diagnosis, therapeutic strategies, impact on quality of life, and other relevant aspects. Further discussed are the intestinal microbiota, its modulation, and dysbiosis in terms of their significance for GRDs. This study highlights important future directions for research as well as controversies regarding the gluten-free diet (GFD), and the current understanding of the underlying mechanisms behind such diseases.

### 1.1. Investigating the Culprit

The term “gluten” is derived from the Latin word *glutinum*, meaning “glue”, in relation to its viscous and elastic properties, giving flour a sticky consistency when mixed with water and salt to form dough. When the starch and water-soluble ingredients of wheat dough are removed by washing with water, what is left behind is actually gluten. The term is also used to refer to the storage proteins in wheat seeds and was originally confined to wheat (*Triticum aestivum*—bread wheat) [6,7,8,9]. However, due to similar toxic sequences in rye (*Secale cereale*) and barley (*Hordeum vulgare*) storage proteins, scientists and the medical community began to use the term “gluten” to refer to all proteins that are involved in provoking GRDs, i.e., the prolamins from wheat, barley, and rye [2,10].

Natural sources of gluten and gluten-related proteins include wheat, rye, and barley, each one having a specific type of prolamin, thought to be antigenic in people with GRDs: gliadin in wheat, secalin in rye, and hordein in barley [6,11,12]. Avenins, a subset of prolamins that make up a small percentage of all oat (*Avena sativa*) proteins, have a different structure from the prolamins previously mentioned [10,13,14]. Although prolamins derived from wheat, rye, and barley have been identified as the proteins responsible for CD, there is growing evidence that glutelins—which have certain amino acid sequences in common with prolamins—may also play a role in the development of CD in predisposed individuals [11]. For example, in the mature wheat grain, gluten makes up about 70–80% of its total protein content and consists mainly of gliadins (a type of prolamine) and glutenins (a type of glutelin). Both are highly heterogeneous due to the multigenic and hexaploid nature of wheat [6,7,15]. Gliadins are a family of alcohol-soluble monomeric proteins and may be further classified as α-/β-gliadins, γ-gliadins, and ω-gliadins. Glutenins, on the other hand, are alcohol-insoluble (acid/alkali-soluble), polymeric, and composed of both high- and low-molecular-weight subunits [7,16]. When hydrated, gliadins and glutenins form a viscoelastic network, making the dough firmer due to protein interactions during dough mixing. These factors make gluten a popular additive in a lot of naturally gluten-free (GF) food items; it can improve their texture and storage qualities, which underlies its significance to the food industry [6,8]. However, gliadins and glutenins also contain repeated amino acid sequences high in proline (>10%) and glutamine (>30%) residues with epitopes that, in individuals with CD, trigger an immunological response [7,16].

### 1.2. Living Gluten-Free: Dos and Don’ts

The GFD is the gold standard therapy for GRDs. A person who needs to adhere to it must eliminate all possible sources of gluten, which include any foods or products that contain gluten, whether it comes from a natural source or is an additive. Gluten can be found in almost all edible products, and even in medicines and cosmetics. However, the term “gluten-free” generally implies that a very small quantity of gluten is believed to be safe to ingest and does not refer to the complete exclusion of gluten [6,17]. It should be noted that 5–50 mg of gluten can be consumed daily per individual by cross-contamination alone. The amount of gluten present in food items is measured in parts per million (ppm), or mg/kg [17,18]. Various safety threshold limits of daily gluten intake have been suggested. While treating CD, after advanced studies, it was concluded that the daily intake of gluten “contaminated” products should be kept below 50 ppm [19]; this would permit the consumption of 500 g of food with 20 ppm gluten without experiencing any negative consequences [20]. Additionally, different countries have different laws governing the requirements and standards for product packaging as well as labeling GF products [4]. The United States Food and Drug Administration (FDA) defines a product, including foods and supplements, as GF [21] if it is either naturally free of gluten, contains no ingredients made from gluten-containing (GC) grains, or is made from a grain that contains gluten but has been processed so that the gluten is eliminated [6], and the product’s gluten content is less than 20 ppm [21]. Countries such as Italy [22], the UK [23], and Canada [24] restrict gluten levels to 20 ppm for GF products, while other nations have stronger limits, such as Spain (10 ppm) [25], Australia, Chile, and New Zealand (3 ppm) [19]. Ever since the European Union (EU) implemented allergy labeling in 2005, gluten cannot be concealed in food products. All foods containing gluten must be included in the ingredients list, with two different levels specified by the EU’s 2009 and 2012 regulations: very low gluten (21–100 ppm/mg/kg) and gluten-free (≤20 ppm/mg/kg) [18,26,27]. In reality, only the gluten-free standard is followed. The estimated safety threshold for gluten that might cause an antigenic response was determined to be greater than 20 ppm gluten, with levels below this threshold deemed acceptable for use in a variety of everyday meals [18].

**Figure 1:**
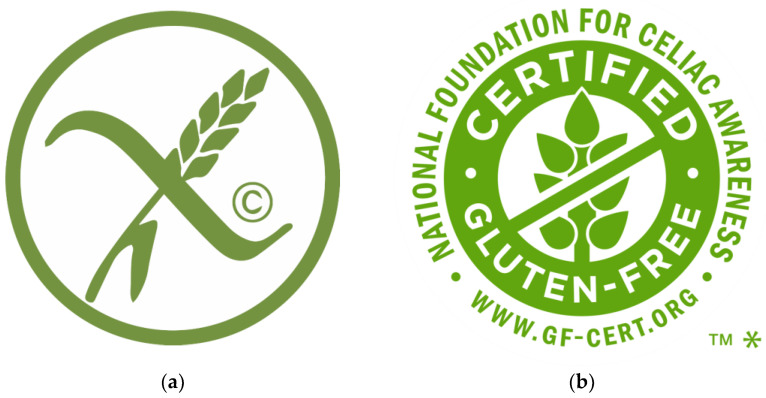
(**a**) The Crossed Grain Trademark (AOECS); (**b**) Beyond Celiac registered trademark used in the United States.

Terms like “Gluten-Free”, “Free of Gluten”, “No Gluten”, and “Without gluten” can be found on packages. Such labeling is essential for empowering customers to make knowledgeable decisions and for assuring patients that what they are ingesting is safe [28,29,30]. When prefabricated foodstuffs appropriately utilize the designation “gluten-free”, it makes it easier for people with such disorders to find items they can safely enjoy [14]. For all customers who are intolerant or sensitive to gluten, nutritional information and well-designed labels are extremely important and useful [31]. Correct labeling of commercially accessible goods to indicate gluten content has already started in Western nations due to rigorous restrictions and growing knowledge of GRDs and their implications. Furthermore, some restaurants designate specific rooms for the production of GF meals, and the precise gluten content of certain foodstuffs is even mentioned in the menu [32,33]. The worldwide mark for GF food, the crossed grain trademark, has a licensing mechanism established by the Association of European Coeliac Societies (AOECS) (Figure 1a). It is restricted to enterprises and groups that fit their requirements [34]. The Beyond Celiac registered trademark (Figure 1b) is being used to sell GF-certified products in the United States [35].

### 1.3. Gluten Contamination and the Oat Controversy

The GFD excludes whole grains like wheat, rye, and barley, and is thus lacking in vital nutrients [14]. While the gluten contained in these grains has minimal nutritional benefits, they are still a convenient and significant source of fiber, vitamins, and minerals [1,6]. For example, wheat supplies energy based on carbohydrates as well as fiber, protein, B vitamins, calcium, magnesium, phosphorus, potassium, zinc, and iron. It is also easy to pack, transport, and store, making it a lucrative option for the food industry [12]. Adding oats to the GFD can help reduce nutritional deficiencies and has a major positive impact on patient health and quality of life, offering whole grain nutritional advantages [10,36] by increasing the intake of amino acids, fiber, minerals, and vitamins as well as the palatability of GF foods [6]. Nevertheless, the safety of oats is a topic of much debate and discussion. There have been concerns about whether or not patients with GRDs, especially CD, may safely eat oats, and it is because there have been conflicting clinical results. A few studies have indicated that certain CD patients may be sensitive to oat proteins [13,14]. On the other hand, other research has demonstrated that CD individuals can tolerate even large GF-certified oat intake with no subsequent risks [10,13]. As previously mentioned, the prolamins in oats (avenins) differ in structure from the prolamins in wheat, barley, and rye. In particular, they do not contain any of the recognized epitopes from wheat, barley, and rye that trigger an immune response [37]. All oat species and kinds have two avenin-specific epitopes, but very few individuals with CD respond to them, making oat intolerance an uncommon occurrence [10]. Pinto-Sanchez et al., in their exhaustive meta-analysis, found no evidence that oat intake—as part of a GFD—modifies the immune response or leads to changes in histological and serological test results, and symptoms in patients with CD, this finding having great importance [13].

Nevertheless, the majority of scientists concur that oats are safe when consumed in their purest form and that contamination with other GC grains must be avoided [6,38]. However, processing oats with grains that contain gluten (while milling or harvesting) can contaminate them with gluten [38]; as a result, oats that are not certified as being GF must be excluded while on the GFD. Oat goods that are not certified GF might also contain gluten [39]. According to Fritz et al., the likely cause of the negative effects linked to oats in CD dietary trials might be because oats that were considered “pure” were really contaminated with wheat, barley, or rye kernels that contained gluten [36]. Thus, patients are encouraged to investigate for information on unpacked foods or to verify the ingredients listed on items that have already been packaged [39]. Patients should be informed about the best ways to choose GF oats, given the freedom to stop consuming them whenever they decide to, and then counseled on whether to keep consuming them in accordance with their histological, clinical, or immunological response. In patients who are more sensitive, oats devoid of gluten contamination may nevertheless trigger an immune response (oat intolerance). Patients should also be informed about other hidden sources of gluten, such as ice cream, processed meats, oats farmed next to wheat, sauces (like soy sauce, marinades, and salad dressings), contaminated kitchen equipment (e.g., toasters, fryers, or kitchenware), and cosmetics (such as lipsticks) [6,17,40]. Even glue and Play-Doh™ can have gluten in their composition [3].

Other food items that are naturally GF can also become contaminated and end up having gluten only as a result of processing in shared areas or storage in locations where GC foods are present [17,19]. The risk of cross-contamination rises when foods are sold in large quantities and kept in open containers, at salad bars or buffets, or in situations where multiple foods are served in close proximity to one another [17]. Furthermore, it was observed that products that are naturally GF but do not have the “gluten-free” tag are considerably more likely to contain gluten [24]. In a study whose purpose was to assess the frequency of gluten contamination in industrialized and non-industrialized GF food items, it was observed that there could be cross-contamination between both types of GF items [20]. For patients that have GRDs, any gluten contamination poses a severe concern since research has proven that the label claims about the presence or absence of gluten can be inaccurate [20], this being demonstrated by multiple researchers [29]. Thus, high attention must be given to cross-contamination. Improved and continuous monitoring, oversight, and regulation of certified GF foods are crucial. It is interesting to see that research demonstrates higher contamination levels in less expensive items, implying higher expenditures for better control [18].

Another thing GRD patients need to keep in mind is that many composite foods, medications, and cosmetics can contain “hidden” sources of vital gluten (VG), a by-product of the manufacture of wheat starch, which is added to naturally GF products to enhance their quality. Toothpaste, mouthwash, and lipstick are just a few examples of oral hygiene and cosmetic items that commonly include VG [39].

### 1.4. Development of Gluten-Free Products

The GF food market is evolving from a specialist sector to a trend in the global food industry as a result of increasing interest toward, and consumption of, GF goods [3,41]. It is well known that gluten is the main protein responsible for dough’s structure, consistency, cohesion, viscosity, and elasticity, affecting the texture and appearance of many baked goods and other foods. As a result, the elimination of gluten presents significant issues, including accessibility, consumer acceptance of organoleptic characteristics, and product safety [28,42]. Vitamins D and B, iron, zinc, calcium, magnesium, and fiber deficits are possible dietary deficiencies that may result when eating GF, and the special qualities of gluten may make it difficult to manufacture high-quality GF goods [43].

As gluten or its prolamin portion, such as gliadin from wheat or hordeins from barley, can cause an immune response, the first aspect to consider when manufacturing GF goods is the exclusion of any ingredient that contains gluten or traces of gluten [28]. Wheat in all its forms, including starch, flour, bran, germ, spelt, semolina, durum, matzo, kamut, triticale, bulgur, and graham flour, should be completely forbidden [19,28,38]. One of wholegrains’ roles, which was recently discovered, is their prebiotic activity for gut microbiota, which is fundamental for the host’s well-being. Grains contribute macronutrients (carbohydrates, fats, proteins) and micronutrients (vitamins, minerals, electrolytes), and are an important source of fiber to the human diet [44]. It is therefore understood that by eliminating the gluten sources from the ingredient lists, there must be a great focus on the nutritional characteristics of the final product in order to ensure the recommended nutrient intake for the individuals who follow a GFD. Naturally, GF food categories include dairy, legumes, nuts, fruits, vegetables, meat, fish, and beans. It is important to note that while gluten is absent from pure wheat grass and barley grass, contamination is possible if the seeds are not separated properly because of the presence of gluten in the actual seeds [6].

Using substitute sources is another factor to take into account when making GF foodstuffs. Wheat typically contains 10–12% protein; therefore, removing it from the diet would mean missing out on an excellent source of protein [6]. As a result, the protein content of the alternative source needs to be taken into consideration. For instance, it is a wonderful idea to replace flour in baking with other sources of protein, such as peas, potatoes, chestnuts, chia flour, egg whites, and whey [43,45]. A newer approach refers to the use of cricket powder in place of flour to provide GF bread with healthy amounts of protein, fat, fiber, and minerals [44]. Other excellent nutrient-dense foods, including soybean, quinoa, millet, chia seeds, sorghum, flaxseeds, beans, chickpeas, teff, yucca, maize, buckwheat, rice, and amaranth, can successfully substitute the gluten sources within the GFD [6,19,46,47]. Unfortunately, they are rarely utilized, partly because of their increased cost and limited availability, even though they may all enhance the nutritional content and palatability of this diet [19].

In addition to being GF, products must have a nutritional profile that is similar to that of GC goods, which is another important aspect to consider and quite difficult to achieve; that is, a nutritional profile that helps to meet the recommended daily allowances (RDAs) of nutrients for the person who consumes them [28]. All patients should be counseled to follow a balanced diet in accordance with the RDAs of nutrients while on a GFD [40]. Though seldom used, mostly due to high costs, a pretty lengthy list of cereals, grains, seeds, legumes, and nuts exists—all of them suitable for use as substitutes for GC ingredients to increase the nutritional value and accessibility of the GFD [19].

It has been suggested that using substitute components with high nutritional and bioactive chemical content, including wholemeal pseudo-cereal flours made from naturally GF grains, may enhance the formulation of GF foodstuffs [48]. Some of the GF and high-protein plants that can be used to make GF flour are found in Table 1. In addition to these, nuts (e.g., almonds, walnuts, cashews), seeds (e.g., chia, flax, pumpkin, hemp), and nut butters are other GF sources that can be successfully exploited for food production [49].

Making sure that the finished product has the appropriate texture and flavor, comparable to GC items, is one of the biggest hurdles in the creation of GF products, e.g., the crumbly, dry texture of GF bread and pasta. The increased susceptibility of GF bread to staleness and mold growth in comparison to GC bread is also an issue. Consequently, an adequate gluten replacement is needed to solve these problems and provide optimal organoleptic characteristics [28]. In trying to successfully replace gluten in the production process, a variety of additives have been used, including protein, hydrocolloids (such as guar gum, xanthan gum, and hydroxypropyl methylcellulose), emulsifiers, fibers, psyllium, enzymes (like transglutaminase), starch, eggs, dairy powder (like skim milk or calcium-fortified caseinate), sourdough, and other materials [43].

Some of the crops listed in Table 1 are used to enhance the aroma, structure, and longevity of baked goods after a biotechnological pre-treatment of their grains or flours, typically including fermentation or sprouting [47]. Although they have not been very successful, dairy and egg components have also been used to enhance the quality of GF bread [56]. Despite the rise of GF food options on the market, those who follow dietary restrictions still report finding it challenging to procure these goods as they typically have short shelf lives, limited availability, cross-contamination risk, high prices and unappealing taste [30,42,48,57]. According to a study conducted by Alencar et al., nearly 93% of the CD patients who participated as responders to a questionnaire said they regularly use GF substitution goods, the majority of which are ready-to-eat foodstuffs, most likely because the home preparation of GF meals can be challenging. These findings highlight how crucial the GF food industry is for people with CD [41].

Food labeled as GF is more expensive and less available than food containing gluten [58], which puts GRD patients’ access to sufficient nourishment at risk, and they experience ongoing food insecurity as a result [59]. Lack of access to healthy food due to its increased cost is a problem that can lead to poor living quality, treatment non-adherence, and socializing issues [42,59]. According to Estevez et al., the market basket for GF items in Chile was 300% more costly, with GF bread costing more than 500% of the price of conventional wheat bread. Furthermore, in the majority of the sites, just 42% of the regular market basket was accessible [60], while a Canadian study discovered that the cost of GF goods might often be 242% more than that of conventional goods [28,30]. In a Mexican study conducted by Calderón de la Barca et al., the average price difference between GF bread and bakery foodstuffs, pasta products, and flours was between 4.3 and 7.5 times higher than that of comparable conventional products; however, some items had price increases of up to 20 times [29]. Lee et al. observed that GF goods were 183% more expensive overall than equivalent wheat-based ones in their research based on the United States marketplace [58]. Therefore, it can be concluded that the GFD can bring a significant financial burden, a fact to be taken into account when developing new products [28]; this diet being linked to a lower quality of life, especially in the social and economic spheres [61].

### 1.5. The Nutritional Deficiencies Linked to a GFD

In later years, there has been a notable increase in people not diagnosed with a GRD choosing to follow a GFD. The general perception that this diet is healthy is a likely contributing factor [62,63]. However, there is no proof that the GFD has any advantages for individuals without proven GRDs and is not advised for everyone due to possible associations with adverse effects [42,64]. Rather, several questions concerning its nutritional worth have been brought forth [18]. GF items made with maize and rice starch have a high glycemic index (GI) and ingesting them may raise the likelihood that an individual with CD may develop a metabolic syndrome (MS). It has also been demonstrated that adding low-fat dairy powders and microencapsulated high-fat powders can make GF dishes more appealing, but the calorie content does rise. Furthermore, GF goods typically lack micronutrients compared to GC items since they are not fortified [65]. It is commonly recognized that the development of MS and cardiovascular disease is facilitated by a high intake of fat, sugar, and salt. Some experts claim that industrialized GF foods may have significant amounts of these; therefore, promoting this syndrome [65], while others state that the actual impact of a GFD on various MS factors is unknown, being a controversial topic [26].

From a nutritional perspective, adequate intake and absorption of macronutrients and micronutrients is necessary for maintaining the structural integrity, proper operation, and energy needs of all tissues [66]. Concerns about the nutritional suitability of the GFD and related products have long existed among consumers and medical experts. Long-term adherence to the GFD may have associated nutritional constraints, even though it has been shown to assist patients with GRDs by promoting gastrointestinal restoration and alleviating symptoms.

Individuals with GRDs tend to buy more prepackaged or processed GF food alternatives, which may be less nutrient-dense than equivalent GC ones [67]. Research has indicated that patients adhering to a rigorous GFD frequently have a range of nutritional deficits due to the restrictive nature of the GFD [2,60,68,69]. Therefore, patient dietary instruction is particularly crucial. It was noted that these individuals’ diets were lacking in balance, being low in fiber and with a larger percentage of calories originating from fat [2,68]. GF grain foods have been shown to be primarily high in fat (total fat and saturated fat), sodium, and carbohydrates, to the detriment of other important nutrients since they are usually made by utilizing refined GF flour or starch and are not supplemented or fortified. To avoid such deficiencies, all patients would need to receive specialists’ help and be closely monitored to ensure adequate nutrient intake [2,6,28,69]. Researchers have attributed the inadequacy of the GFD mainly to poor dietary habits, and they concur that appropriate monitoring, liaison with dietitians, and nutrition advice are critical in order to guarantee that individuals adhering to the diet do not face any supplementary risks to their health. Food fortification would also need to be taken into account [68,69,70].

**Figure 2:**
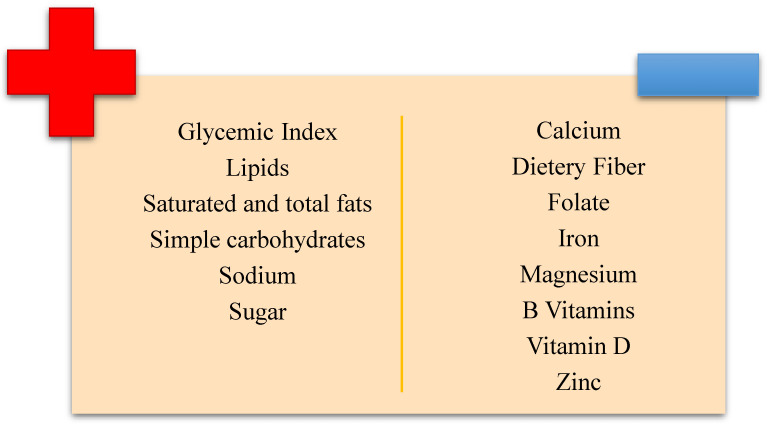
Nutritional deficiencies and excesses associated with GFD.

The GFD has been found to often be low in ions and vitamins, such as calcium, iron, zinc, magnesium, B and D vitamins, and others, suggesting that the RDAs are often not reached for these nutrients [28,68]. Many variations in the micronutrient analytical content (minerals and vitamins) between GF and GC goods are identified [44]. The main nutritional deficiencies and excesses associated with GFD can be seen summarized in Figure 2.

The quantity and quality of ingredients used in GF goods, together with the food processing techniques employed in their manufacturing, all affect their GI, which has been shown to be significantly increased compared to their GC counterparts. Additionally, this may increase the risk of obesity and glycemic control loss in individuals with type 1 diabetes who are not well-informed [2,68]. Moreover, Remes-Troche et al. observed in their study that following the GFD for six months resulted in a twenty percent rise in obesity and MS [71].

The GFD is associated with small amounts of iron found in everyday food, therefore patients should be advised to eat iron-rich foods [72] (e.g., red meat, organs from cattle, seafood, and poultry, as well as non-animal foods like spinach, lentils, and leafy green vegetables [73]), iron-fortified grain foods, GF foodstuffs fortified with iron, and even iron supplements, if a physician prescribes them [72]. Body iron stores should be checked periodically [72].

In their study, Cyrkot et al. concluded that GF products often contain less folate than foods containing gluten, making it more difficult to achieve the RDA. This raises the need for processed GF foods to be subject to a mandatory folate fortification policy. Many foods, such as legumes, spinach, kale, romaine lettuce, broccoli, and eggs, as well as seeds and nuts, are folate-rich and recommended to be included in the GFD [67]. However, it is suggested that GF goods enhanced with folate might help to avoid the shortages that have been reported [44]. It has also been demonstrated that there may be serious mineral deficiencies linked to the GFD, and it was discovered that over 10% of patients undergoing this type of restrictive diet had inadequate consumption of certain minerals, specifically magnesium, calcium, zinc, and iron for both sexes [68,72]. As a result, it is important to promote continuing surveillance of patients and dietary counseling on nutrient intake.

Vitamin D and calcium deficiencies are common side effects of a GFD. Research indicates that using vitamin D supplements while on a GFD helps to regulate calcium levels, relieve symptoms associated with osteomalacia, and avoid additional bone loss [74]. Given that most individuals with GRDs consume less calcium and vitamin D than the RDA [72], it is critical to identify these deficiencies as early as possible. This is likely caused by the continued evasion of lactose-containing dairy products, particularly since CD and lactose intolerance are frequently associated, as well as the absence of calcium and vitamin D fortification in GF products [6]. Therefore, supplementation is recommended in patients with low serum levels, those with loss of bone mineral density, or those who cannot reach the RDA [6,74].

Numerous studies have shown that the GFD is frequently characterized by a reduced consumption of dietary fiber in comparison to a typical GC diet due to a drop in the dietary intake of grain products [68,72], which can exacerbate constipation in GFD patients [72]. GF products are often prepared using refined flours or starches that have a reduced amount of fiber [68,72]. It is well-recognized that eating enough dietary fiber has significant health advantages, including lowering the risk of diabetes, cardiovascular disease, and colon cancer [68].

## 2. Celiac Gluten Intolerance or “Celiac Disease”

### 2.1. Definition, Physiopathology, and General Presentation of the Disease

Digestive enzymes (chymotrypsin, pepsin, and trypsin) in the gastrointestinal tract proteolyze gluten before it reaches the duodenum. This results in gluten-derived peptides (also known as “gluten immunogenic peptides, GIPs”), which are resistant to further digestion since they contain a significant amount (>10% of the total primary protein) of proline and glutamine residues. This is considered the cause of improper gluten digestion [7,8,16,75]. After traversing through the intestinal lumen and entering the lamina propria, these intact gluten peptides are post-translationally processed by tissue transglutaminase type 2 (tTG2) [7,32]. Human leukocyte antigen (HLA)-DQ cell surface receptors allow activated gluten peptides to bind to potential antigen-presenting cells quickly. The process of deamidating glutamine to glutamate, which is essential for transforming natural gluten peptides into powerful antigens, is catalyzed by tTG2 [7], leading to the improvement of binding efficiency to HLA-DQ2 and HLA-DQ8, which preferentially accept peptides with negatively charged residues at anchor sites [63]. This results in the release of pro-inflammatory cytokines and chemokines by CD4+ T helper cells and the cytotoxic activity of CD8+ T cells that are linked to mucosal damage, which consequently causes the onset of symptoms associated with CD and chronic cellular inflammation [7,63]. This causes the humoral immune system to target gluten and the autoantigen tTG2, which in turn causes a T-cell-mediated immunological response upon contact with gluten and a malabsorptive enteropathy, also known as “gluten enteropathy” [6,76]. Thus, it has been shown that these GIPs may cause villous atrophy, intraepithelial lymphocytosis, and crypt hyperplasia by initiating an inflammatory cascade [6,16]. Gluten-specific CD4+ T helper cells from the intestinal mucosa are precisely activated by nine amino acid epitopes within the immunogenic peptides that are recognized by HLA-DQ2 or HLA-DQ8 receptors [6,16,63]. There are hundreds of distinct immunogenic peptides that range in length from 9 to 33 amino acids that have been discovered; however, the immunodominant “33 mer” peptide that is produced from α2-gliadin has been investigated the most [16,76], being resistant to digestion across the whole gastrointestinal system and recognized as the major inflammation trigger in people with CD when they are exposed to gluten [75].

**Figure 3:**
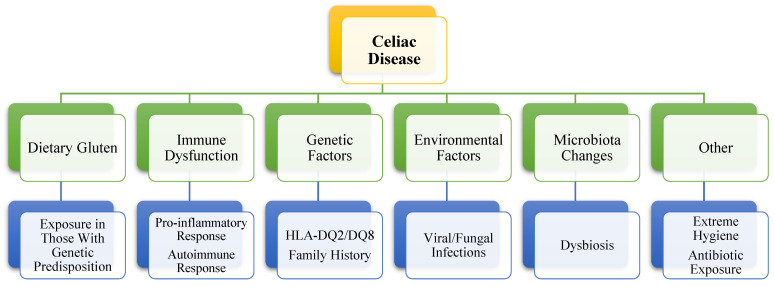
Examples of potential causes and contributing factors to CD.

Consequently, CD can be described as a chronic enteropathy, an autoimmune disease mediated by the small intestine immune system that is brought on by dietary gluten exposure in those with genetic predisposition, its pathogenesis including autoantibodies against endomysium (anti-EMA), tissue transglutaminase type 2 (anti-tTG2), and deamidated gliadin peptides (anti-DGP) [40,77,78]. This medical condition is linked to intraepithelial lymphocytosis, mucosal inflammation, duodenal villi atrophy, and crypt hyperplasia, which cause malabsorption and other gastrointestinal and extraintestinal symptoms [17,79]. Just 1–3% of people who carry the HLA-DQ2 or HLA-DQ8 (rarely HLA-DQ7) genotype, truly develop the condition, despite these haplotypes being prevalent in up to 40% of the human population, suggesting that they are essential but not sufficient to cause CD, different environmental factors also playing a significant role [63,77,78,80,81,82]. Originally thought to be a very uncommon gastrointestinal disorder that mostly affected young white children, it was demonstrated that CD can occur at any age and burden people of any race, and it entails a strict GFD to be followed for life [32,77]. Some of the potential causes and contributing factors to CD can be found in Figure 3.

Dutch doctors were the first to identify gluten as the cause of CD, also known as celiac sprue or idiopathic sprue, in the late 1940s [83]. Samuel Gee initially reported CD in 1887 [77]. Even though Willem Karel Dicke did not propose wheat as a potential cause until 1941 [77], he was the first to develop the GFD during World War II [3]. A study from 1957 provided further evidence that gluten causes adult-onset CD and, as a result, the way that this (once-“idiopathic”) disease was treated in the United States was significantly modified [83].

The understanding of the epidemiology, clinical presentation, pathophysiology, and management of the disease has changed since the initial description [77]. There is strong evidence that CD is an autoimmune disease triggered by the ingestion of gluten-related proteins present in wheat, barley, and rye in genetically predisposed individuals [40,84]. This disease affects 1–3% of the general population, with slight differences in prevalence between countries and continents, races, and ethnicities [16,81,84,85]. The distribution of clinical characteristics has changed, and illness incidence has increased as a result of the growing knowledge and the emergence of serologic testing. It was initially believed that CD mostly affected people in Northern and Western Europe; however, it is now known to affect people all over the world [84]. According to King et al., women are more likely than males to be diagnosed with CD [85], and yet the incidence in men is steadily rising, and it was observed that the chances are 2–3 times higher in women than in men to develop this condition [86,87,88]. Compared to adults, children are twice as likely to obtain a CD diagnosis [85,86].

Since CD typically first manifests during infancy or childhood, it is crucial to examine early life experiences in order to determine the potential causes [82]. With a reported frequency of 1–3%, pediatric incidence estimates for CD are now rising in the United States and Europe [81]. In genetically predisposed children, higher gluten intake during the first five years of life has been associated with statistically significant elevated chances of developing CD [82,85]. At about two years of age, a child’s consumption of more than 2 g/day of gluten—roughly one slice (35 g) of white bread or one plate of cooked pasta (150 g)—was associated with a considerably higher risk of this negative result [82]. Liu et al. discovered that throughout the first ten years of life, there is a significantly increased incidence of developing CD symptoms [81].

Patients with CD can often present with nutritional deficits, another autoimmune illness (35% of patients), or a family history of CD in those who have the disease [3,88]. There is a big genetic component to CD, and the epidemiologic data shows that it can afflict up to 20% of first-degree family members of CD patients [89]. Type 1 diabetes, alopecia areata, dermatitis herpetiformis, Down syndrome, Williams syndrome, Turner syndrome, Sjögren syndrome, autism, selective IgA deficiency, severe food allergies, psoriasis, hepatic autoimmune disease, Addison’s disease and rheumatoid arthritis are among the disorders that have been significantly linked to CD [15,85,87,90]. Numerous studies provide insight into risk factors that promote the development of CD. Notably, there is a real rise in CD prevalence [3] outside of diagnostic advancements, most likely as a result of environmental conditions [85]. According to Gnodi et al., a change in the immunological response is a crucial phase in the predisposition and pathogenesis of CD. Recent research has focused on the possible role of epigenetics in CD since environmental variables can also create epigenetic alterations that influence inherited features. Although epigenetics has received a lot of attention lately, its role in CD is still not fully understood. A lot of focus is placed on the further predispositions to CD, the role that epigenetics plays in the development of CD complications, the pathogenic pathways that are influenced by epigenetic factors, including microRNAs, and the potential application of epigenetic profiling as a biomarker to distinguish between patient categories [80]. These findings point to further research that is much needed in this direction.

A pro-inflammatory innate immune response driven by gluten, smoking, an improper adaptive immunological response, living at northern latitudes (north of the 40th degree parallel), changes in intestinal microbiota (e.g., the lack of *Helicobacter pylori* colonization), pediatric viral infections (e.g., reovirus, rotavirus, norovirus), fungal infections (e.g., *Candida*), genetic predisposition, extreme hygiene that leads to reduced microorganism encounter during early life (resulting an a hyperactive immune response developed later in life), to antibiotic exposure in the first year of life and loss of intestinal barrier function, cesarean delivery of babies, formula feeding, all appear to be other important risk factors that establish the environment necessary to trigger an anti-gluten reaction [81,85,87,91,92,93]. There is no strong evidence to suggest that the timing of dietary gluten introduction, the length of nursing, or gluten consumption during pregnancy increases a child’s chance of developing CD, but these are still variables to take into account [81,82,91]. According to Aronsson et al., in children who are genetically susceptible, consuming more gluten during the first five years of life has been linked to an elevated risk of CD [82]. Numerous theories (such as the fact that modern gluten is of a different quality than it was in the past or that technical advancements have resulted in the creation of new grain variations) have not been proven to be true, and it is still unknown what exactly causes the risk of developing CD [94]. Moreover, the epidemiological finding that comparable “epidemics” are documented for other autoimmune disorders throughout Western countries raises the possibility that environmental variables other than gluten may be involved [81,94]. In Western nations, the number of cases of CD is still rising. In the United States, the median age of diagnosis for CD is between 40 and 50 years old [90], and between 1975 and 2000, CD prevalence rose five times for unexplained causes [90,94].

CD has well-recognized nutritional complications, such as iron-deficiency anemia or various micronutrient deficiencies, along with reproductive-type complications (late menarche, early menopause, amenorrhea, infertility, increased risk of spontaneous abortion, reduced intrauterine growth, increased risk of premature birth or infant death at birth, recurrent miscarriages), and children with CD may have developmental limitations such as short stature and growth retardation [15,87]. Patients with CD present higher rates of mortality (1.4 times increased risk [19]), morbidity, and an increased risk of developing even autoimmune diseases or malignancies, including adenocarcinoma of the small intestine (30 times increased risk [19]), esophageal cancer, or non-Hodgkin’s lymphomas (2–4 times increased risk [19]) [7,15]. However, it should be noted that the risk for complications and mortality is reduced by following a GFD [15,87].

### 2.2. Symptomatology

CD was originally viewed as a disease which causes malabsorption due to diarrhea and results in limited growth and development in children. It was thought that the disease developed as soon as gluten was added to the diet, and the severity of the immune reaction determined when the symptoms appeared. On the other hand, the advent of highly sensitive and specific non-invasive tests has made it possible to measure the prevalence of CD more accurately, identify people and populations at risk, and contribute to the understanding that CD is a systemic autoimmune disease with an onset that can happen at any age and express symptoms that are extraintestinal, gastrointestinal, or both [77,95].

**Figure 4:**
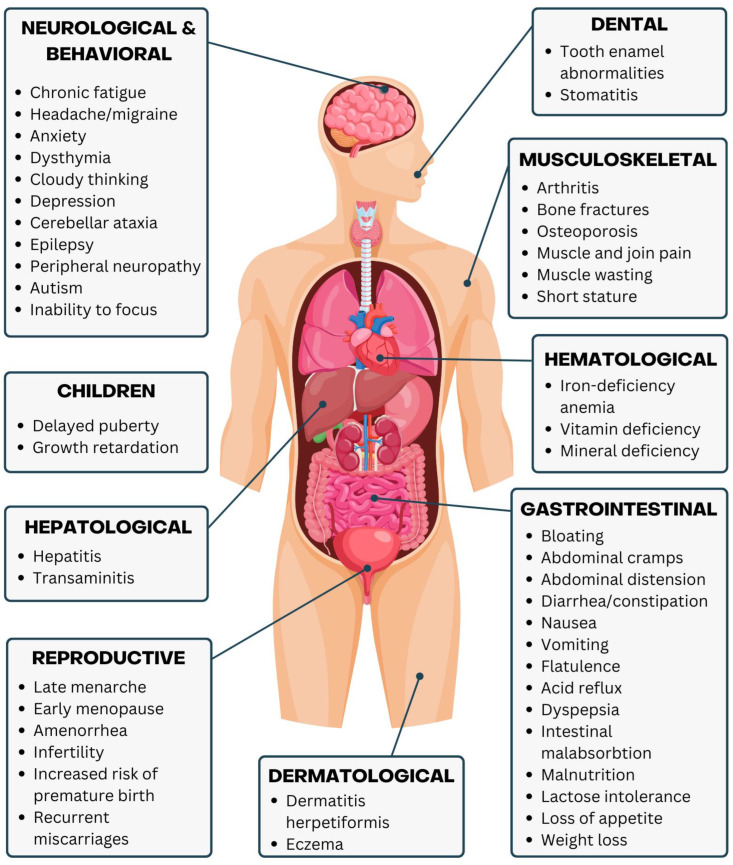
Examples of signs and symptoms in untreated CD patients.

Almost every bodily system can be affected by CD [88], as seen in Figure 4. A characteristic constellation of gastrointestinal symptoms, such as diarrhea, constipation, vomiting, bloating and enlarged abdomen, flatulence, nausea, IBS-like symptoms, acid reflux, dyspepsia, intestinal malabsorption, intolerance to lactose (can occur in 50% of patients at diagnosis and ought to disappear after intestinal healing) and other carbohydrates, stomach cramps and loss of appetite, can appear. These symptoms might be accompanied by those who are extraintestinal, such as decreased quality of life, anemia, chronic fatigue, weight loss, short stature, mineral and vitamin deficiency, neurological issues (some arising from B vitamin deficiency), osteopenia (due to vitamin D and calcium deficiency), and even dermatological (e.g., dermatitis herpetiformis, eczema) and respiratory symptoms [15,87,90,92,96]. Atypical presentations of CD are frequent and might include minor gastrointestinal symptoms along with iron-deficiency anemia, pancreatitis, tooth enamel abnormalities, finger clubbing, stomatitis, infertility, and other reproductive issues (e.g., unexpected abortion), osteoporosis, osteomalacia, muscle wasting and pain, arthralgia, arthritis, transaminitis, hepatitis, and neurological symptoms. CD is linked to neuropsychiatric illnesses such as headaches, migraines, peripheral neuropathy, cerebellar ataxia, dysthymia, anxiety, epilepsy, inability to focus, cloudy thinking, autism, behavioral issues, and depression. Neuropathies can also result from chronic malabsorption-induced deficits in vitamins E and B. The diagnosis of CD is complicated by the existence of asymptomatic individuals (about 21%) and those whose symptoms may consist of mild indicators, such as persistent fatigue or other extraintestinal symptoms [15,85,87,90].

The most common presentations in infants and young children under five years old include diarrhea, growth retardation, failure to grow and thrive normally, and distension of the abdomen. Abdominal discomfort, constipation/diarrhea, weight loss and bloating are typical symptoms reported by older children [15,87]. As the age of diagnosis increases, symptoms become less gastrointestinal-focused and milder, and they tend to be more extraintestinal. Even in the absence of symptoms and gastrointestinal discomfort, teenagers might have delayed puberty, modest height, neurological conditions, and anemia [15].

It is also critical to note that the symptoms can vary widely throughout people, nations, climates, and other factors. They might appear right away after ingesting GC foodstuffs or take up to 48 h to manifest themselves [96]. It is important to acknowledge that when patients begin a rigorous GFD, these signs and symptoms can usually be reversed [87]. It is, therefore, of crucial importance to diagnose CD as early as possible and to properly implement a GFD.

### 2.3. Diagnosis

CD is diagnosed by taking into account clinical characteristics, serology, histology, and genetics [5]. As previously mentioned, the clinical presentation of CD is highly variable, encompassing non-intestinal manifestations such as osteoporosis, dermatitis, and anemia, as well as traditional intestinal symptoms like diarrhea and inability to develop properly. In many instances, this varied clinical picture may impede the timely identification of the condition [80]. First-degree relatives of CD patients, as well as those with autoimmune diseases or other conditions that increase the risk of developing CD, should undergo screening tests [90]. Untreated CD raises the risk of consequences such as osteopenia, infertility, and cancer, in addition to malabsorption, diarrhea, poor development in children, and stomach discomfort. Therefore, the medical community has remained keenly interested in making an early diagnosis of small intestine enteropathy, even prior to the onset of the signs and symptoms [87].

**Figure 5:**
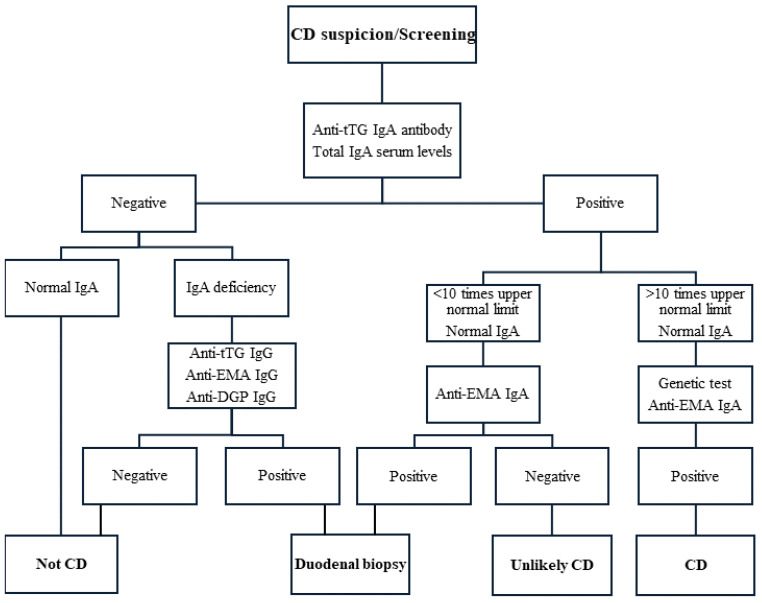
Suggested CD diagnosis scheme for patients on a GC diet.

In order to verify the diagnosis of CD, antibody serology tests (anti-tTG2, anti-EMA, and anti-DGP antibodies), small intestine biopsies, and occasionally genetic testing are performed [5,40]. A methodical assessment utilizing IgA anti-tTG2 antibodies and a small intestine biopsy is usually undertaken for the precise and reliable diagnosis of CD [97]. Regarding diagnostic criteria, there is considerable dispute about whether a small intestine biopsy is required. In patients who have abnormal serologic test findings or if there is a substantial clinical possibility of CD in the absence of positive serologic test outcomes, endoscopy is usually utilized to confirm the diagnosis by taking biopsy specimens of the proximal small intestine [90]. According to Fuchs et al., three factors indicate that endoscopy can be avoided: (a) an anti-tTG2 antibody level more than ten times the upper limit of typical levels, (b) a positive anti-EMA antibody test result, and (c) CD-compatible genotype without symptomatic requirement (since genetic tests could be used to exclude CD in ambiguous cases). Reliable non-invasive CD diagnosis can be confirmed for individuals who test “triple positive”. Using the “triple criteria”, 33% of newly diagnosed cases of CD may be identified, avoiding the need for an intestinal biopsy. In addition to being more convenient for patients, fewer endoscopies may result in significant healthcare cost savings since skipping the biopsy is thought to save up to 95% of diagnostic costs. Fuchs et al. concluded that doing endoscopies on all “triple criteria” positive patients is not necessary [98]. Figure 5 summarizes a suggested CD diagnosis plan that should be taken into consideration for patients on a GC diet.

The European Society for Paediatric Gastroenterology, Hepatology, and Nutrition (ESPGHAN) and the American Gastroenterological Association agree that a biopsy-free protocol for CD diagnosis is possible for patients with significantly elevated serum tTG-IgA antibody levels (>10 times the upper normal limit) [63,99]. The likelihood of a positive result for CD is about 100% when such elevated serum levels are paired with a positive anti-EMA antibodies test in a second blood sample [99,100]. Because false-positive cases of CD are common in adults, the serological test positivity alone is inadequate to confirm the diagnosis. Therefore, before beginning a lifetime GFD, it is essential to validate the condition with a small intestine biopsy in addition to the serological tests [100]. Moreover, confirmatory biopsies are still advised according to North American norms. The use of endoscopic, laboratory, and clinical techniques is implied in the diagnosis process [101]. In the adult population, the most common clinical protocol and the ‘gold standard’ diagnostic procedure is the histopathological evaluation of small intestinal specimens; it can be done in addition to serological testing [88,92]. Children must have symptoms and indications of CD that are unmistakably indicative of the disease, a level of anti-tTG2 more than ten times the upper limit of normal levels, a positive anti-EMA antibody test acquired at a period different from the anti-tTG2 antibody dosage, and an HLA genotype consistent with CD in order for the endoscopy to be avoided [77,92]. If the anti-tTG2 antibody titers are less than ten times the upper normal limit, a biopsy is still necessary to establish a CD diagnosis [92]. Videocapsule endoscopy is a non-invasive, useful, and alternative technique for examining the intestinal mucosa [92,102].

The most effective methods to test for CD, as seen in Figure 5, can be controversial when used in individuals who have already been GF for a significant period of time [63] since one drawback of the existing antibody-based diagnostics is that they are not very specific to diagnose individuals who are currently adhering to a GFD [92]. Many individuals begin a GFD prior to the necessary diagnostic testing; therefore, under such circumstances, the length of the GFD and the extent to which gluten has been actually eliminated influence how accurate diagnostic serologic testing or biopsies are after a gluten withdrawal. It is acceptable to conduct serologic investigations for CD at the first appointment, even if the patient reports adhering to a GFD, since histologic abnormalities frequently take a long time (even multiple years) to come back to their normal state on a GFD [90]. Since serologies and histology in CD patients following a GFD may seem normal, another form of testing is undergoing a gluten challenge (unless consuming gluten causes major symptoms), which refers to the consumption of 3–10 g of gluten (1–2 slices of whole wheat bread) per day for 2–8 weeks [15]. The symptoms are then closely monitored, and if they do not occur, the gluten challenge should be continued for at least three to six months. After this time, duodenal biopsies should be performed [90].

Determinations of HLA-DQ2 and HLA-DQ8 are also helpful when histological and serological results disagree [77], considering that CD patients nearly always have HLA-DQ with DQ2 and DQ8 heterodimers. HLA typing, a molecular-genetic test, could be used to rule out CD in patients who follow a GFD prior to testing, who do not attempt a gluten challenge, and have negative serologies and equivocal histological results (Marsh 0 or 1—Table 2). However, this typing should not be carried out for routine CD evaluation [15]. Villous atrophy, crypt hyperplasia, and intraepithelial lymphocytic infiltration are caused by CD [92], the first being detected via a small bowel biopsy, which can conclusively diagnose CD [77]. The modified Marsh-Oberhuber grading system, shown in Table 2, is the most often used histological categorization for assessing the alterations linked to CD [15,75,92]. Three subcategories (3a, 3b, and 3c) are included in Oberhuber’s subdivision of Marsh 3 type, which aids in the interpretation of the histological presentations of flattened mucosa [103].

Patients with normal intestinal histology (grade 0 or 1), positive anti-EMA and anti-tTG antibody tests, and specific CD genotypes are considered “potential” CD patients (10% of CD individuals are in this category). They can have symptoms or be asymptomatic, nonetheless, and GFD is advantageous for them. It should be understood that asymptomatic CD and potential CD are different phenomenon because this “potential” variety of CD remains unaffected or mildly inflamed at the intestinal mucosa level, in contrast to all other types of CD, which are distinguished by abnormalities in this region of the body [17,104]. Less than 2% of CD patients test negative for serological markers, making them “seronegative” CD individuals, a rare subgroup that mainly consists of elderly people. In these instances, the diagnosis is intimately linked to the intestinal histology’s finding of villous atrophy. Conducting a genetic test for CD is still a crucial step since a negative result definitely rules out the illness and encourages medical practitioners to look for alternative reasons for villous atrophy (e.g., autoimmune enteropathy, intestinal bacterial overgrowth). A seronegative CD may be verified one year from the start of a GFD since this is an appropriate period to show remission in both symptoms and histology [105,106].

As stated before, CD is not just a pediatric condition, and it can occur at any stage of life, with the average age of diagnosis being 40 years, but it could be identified or triggered even at a geriatric age since studies have observed adulthood-related reduction in gluten tolerance [7,84,87]. Complex testing should be performed in patients exhibiting signs and symptoms suggestive of CD, particularly if they have first-degree family members with this disease [15,81]. The first screening test for CD that is advised for everyone, regardless of their age, is the evaluation of anti-tTG2 IgA antibodies [3]. Since IgA deficiency is a rare but significant condition, there is a chance that patients with both abnormalities will have false negative results when testing for CD. To address this, both IgA anti-tTG2 antibodies and the total IgA level should be measured, and the total IgA level should be determined in patients who have a negative antibody test but for whom strong CD suspicions exist [99]. In order to serologically identify CD in this specific patient group, it is also advised to measure the levels of IgG anti-EMA (which have sensitivity and specificity similar to IgA anti-tTG2), IgG anti-tTG2, and IgG-DGP antibodies [3,92,99].

According to Hosseini et al., CD patients are at an increased risk of obesity, and approximately 50% of adult patients are obese. However, as obesity is even more common in children with CD, it is important to screen obese children for CD. Commercially accessible GF meals have a high-calorie content, which may lead to weight gain [43]. The length of the GFD was correlated with a rise in the body mass index (BMI), indicating that weight management counseling is a crucial component of dietary instruction and follow-up treatment for CD [3]. Moreover, intestinal villi destruction can cause impediments to food absorption and digestion, which can contribute to obesity [43].

### 2.4. Nutritional Deficits Associated with CD

Deficits in electrolytes, vitamins, and minerals are common (over 90% of patients) among untreated cases of CD due to inadequate dietary intake, malnutrition, malabsorption, and maldigestion, with potential repercussions for other bodily systems [107]. The small intestine is the target of the inflammatory process, which damages the villi gradually and causes malabsorption of minerals, including iron, folate, magnesium, calcium, zinc and fat-soluble vitamins [30,49,108], resulting in crippling effects including anemia, osteoporosis (leading to an increased risk for bone fractures), hormonal imbalances, cancers, neurological conditions, and impaired development [108]. At diagnosis, several micronutrient deficiencies are prevalent, and they often continue even when a GFD is followed. In people with CD who have over two years of strong GFD adherence, incidences of iron and zinc inadequacy are up to 40%, vitamin B12 deficiency 30%, and a folate deficit of 20% were described [49]. This may indicate that inadequate food intake rather than active disease may be the primary cause of nutritional shortcomings, and recent research findings support this hypothesis [109,110].

Iron, B vitamins, and fat-soluble vitamins (A, D, E, and K) are all predominantly absorbed in the proximal small intestine; loss of these nutrients may occur due to CD [90,111], and their deficit may be present in a quarter of children diagnosed with CD [77]. Nutrient deficiencies, such as those in zinc, folate, and vitamin B12, can be present also in adults with CD [77]. The levels of certain minerals, such as magnesium, copper, zinc, and selenium, might also be low based on the severity of the condition and the food that is consumed. In addition to a GFD, vitamin and mineral replenishment is advised until the digestive tract recovers and earlier deficiencies are restored [90]. In recently diagnosed individuals, particularly those who have inadequate vitamin D levels, the density of bones needs to be evaluated [90]. Nutritional indicators should be assessed as soon as a patient is diagnosed with CD, and reevaluation of erroneous test results ought to occur no later than a year following the introduction of the GFD [77]. With strict and ongoing dietary therapy with a GFD, the majority of deficiencies can be corrected [108]. In cases when the GFD alone or compliance is problematic, and they cannot correct the nutrient imbalances, intravenous delivery of supplements may alternatively be used [112]. It was reported, in accordance with the guidelines of the National Institute for Health and Care Excellence (NICE), that certain patients with CD might require extra vitamins and minerals despite following a GFD, primarily in the initial phases following diagnosis. It was suggested; however, that this should be determined through a suitable continuous follow-up procedure and that the integration of supplements into therapy should be started after an extensive examination [112].

### 2.5. Therapy and Impact on the Quality of Life

As the most successful therapy, a lifelong GFD is strongly advised for anyone with a CD diagnosis, according to all CD care guidelines [32,63,113,114]. Being aware of the illness has a detrimental impact on one’s quality of life in terms of health; this impact extends beyond patients to include parents, other family members and even friends [32]. In addition to being expensive, full adherence to a GFD is viewed as a hardship since it restricts social and work/school activities, food choices, and could adversely affect patient health [19,32].

The culinary arts are deeply entwined with world cultures and serve as a central theme in many societal structures. Social gatherings centered around a variety of cuisines are frequently held to commemorate life milestones, marriages, and anniversaries. Because food has such a powerful influence on daily life, strict dietary restrictions may lead to instances of social exclusion and dissatisfaction [30]. Strict adherence to the GFD is challenging, in part because it makes CD patients more socially isolated [43], especially when dining out, traveling, attending social gatherings, or consuming food that has been prepared by others. Elderly people, the uneducated, those with mental or psychiatric impairments, and people with inadequate financial resources are among the groups that suffer the most from a GFD [3].

Studies on intestinal biopsies have demonstrated that while some patients can achieve complete normalization of the mucosa within six months of beginning a GFD, most patients will typically need a longer period of time—up to 2 years or more—before repeated biopsies reveal a fully healed and recovered intestinal mucosa [115]. After five years of beginning the therapy, more than 60% of patients experience complete mucosal healing [17]. Even when adhering to a rigorous GFD, up to 30% of CD patients experience persistent or recurrent symptoms over time [116], usually due to gluten ingestion (intentional or by mistake, e.g., consuming gluten via contamination) or other associated pathologies [3], but only up to 1.5% have refractory CD, which is unresponsive to treatment, still presenting typical CD serology, signs (such as villous atrophy) and symptoms while under GFD for at least one year [87,107,116]. Nonetheless, most patients may ultimately see mucosal healing since the rate of continuous villous atrophy declines over time on the GFD [3]. A rigorous GFD, which promotes intestinal repair and symptom reduction for most people, is necessary for managing CD. Both extraintestinal and gastrointestinal symptoms can arise from not following the GFD, leading to a decreased quality of life; the most frequent ones are exhaustion, food cravings, physical discomfort and pain, and poor energy [117]. Important nutrients cannot be absorbed as a result of the inflammation linked to CD, although this can be mitigated by adhering to the GFD [6,17]. Unfortunately, adhering to this kind of limited diet is sometimes difficult and may lead to possible nutritional imbalances. Thus, in order to enhance the health state of individuals with CD, dietary care and supervision is essential [40].

The potential ineffectiveness of the GFD as a CD therapy has sparked an abundance of interest in the creation of innovative treatments. Notwithstanding this zeal, no medication has as of yet shown effectiveness in a Phase 3 clinical trial; therefore, there is currently no licensed medicine for CD; however, there are several non-dietary therapy options that are being explored [118]. The use of probiotics is justified by research showing that the duodenal microbiome of people with CD is dysbiotic both before and after the illness manifests itself. Probiotics have been suggested as a potential therapy for the persistent symptoms that many CD patients experience; their potential involvement in treating CD in conjunction with the GFD is yet unclear despite encouraging pilot research [114].

### 2.6. Adherence to Therapy and Follow-Up

Because CD is linked to higher morbidity and mortality, routine medical check-ups are seen as an essential part of patient management. For patients, symptom relief, decreased risk of complications, and achieving the best possible quality of life are the main objectives of treatment, and for physicians, disease remission also means the recovery of the intestinal lining [119]. Although there is no cure for CD, the GFD ultimately serves to restore the damaged intestinal lining and reduce (and, if possible, completely eliminate) symptoms; therefore, it is very important to educate and motivate patients to follow the diet accordingly [90,111]. Nonadherence is widespread, particularly in young people, and even while the symptoms usually go away in a relatively short period of time (a few days or weeks) after treatment starts, immunologic sensitivity to gluten does not go away; therefore, if gluten is again introduced into the diet, usually the negative repercussions will have a fast return [90]. Retrospective United States research discovered that the death rate of patients with untreated CD was four to five times higher than that of a control group undergoing a GFD. High gluten intake prior to, as well as following diagnosis, has been linked to an elevated likelihood of death from CD [90]. Community support groups, particularly those connected to medical specialists or nutritionists, may help raise awareness of the importance and characteristics of the GFD, as well as the disease and its related complications. This could lead to better adherence to the GFD and nutritional monitoring [111].

**Figure 6:**
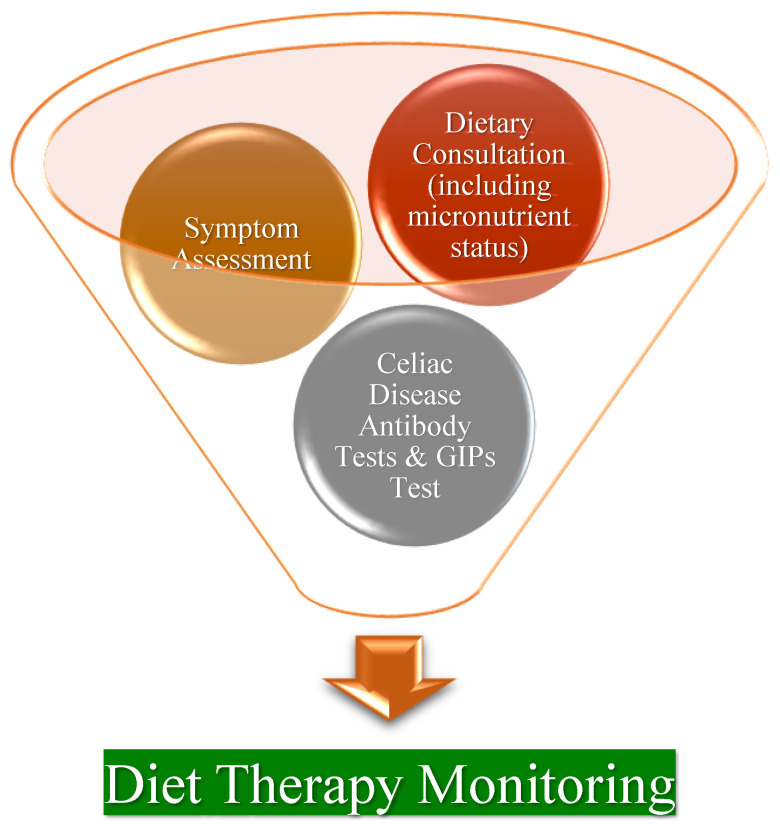
Steps to follow when monitoring the GFD adherence and compliance in CD patients.

Dietary adherence must be evaluated, and the diagnosis verified by an objective reaction to a GFD. The main steps used in this process are displayed in Figure 6. Usually, enough information is available to assess the response to diet therapy from clinical, laboratory, and serologic testing. A registered dietitian and a physician should be on the same medical team and monitor patients on a regular basis, looking into their dietary habits and history (via interview or patient self-reports), laboratory tests (such as the determination of nutrient status and CD serology), symptoms evolution, and endoscopy result (if necessary) [77,90,119,120]. It is crucial to recognize that up to 40% of patients receive no dietary advice from specialists and frequently withdraw from primary care follow-ups [121]. As a consequence, not all patients prepare their meals correctly or choose foods that are acceptable for them. Individuals with CD who have just received a diagnosis may benefit greatly from dietary advice [57]. Normally, it is advised to follow-up for life, and annual follow-ups should start as soon as the condition is stable and the patients are able to maintain their diet without any issues. The primary goals of a CD follow-up are to look for mucosal healing and the return of patients to normalcy, as determined by the absence of symptoms, according to the British Society of Gastroenterology [122]. Thus, after initiating GFD, CD-specific autoantibody (anti-tTG2, anti-EMA, anti-DGP) measurements should be conducted approximately every six to twelve months until they eventually stabilize, as per current recommendations [77,90]; it is important to emphasize that these results are not well correlated with symptom and intestinal status [75]. After a year of medical nutrition therapy, some specialists advocate for another small intestine biopsy; however, others disagree, arguing that a second biopsy is not necessary for managing CD in most patients [122]. Repeated endoscopy with duodenal biopsy should be undertaken if, after six to twelve months of therapy, antibodies still remain high or the biopsy results turn positive [90]. In individuals exhibiting symptoms, nutritional deficits, or declining bone mineral density, a histological reevaluation may be necessary to ascertain if these problems are related to ongoing CD activity [119]. In individuals who have stabilized clinically and whose celiac serology has returned to normal, repeated endoscopy is sometimes advised one or two years into a GFD in order to verify mucosal repair, particularly in individuals who had originally severe manifestations or are older than 40 years of age [113,119].

Regarding how often and what type of monitoring individuals with CD should have, how to assess their adherence to the GFD, and how to monitor their therapy precisely, there are no official guidelines in place [75,111]. New guidelines dealing with CD and other GRDs are necessary, taking into consideration that the currently available international guidelines, both for children and adults, are outdated [113]. An annual assessment comprising laboratory testing (such as serological nutritional characteristics and serum IgA anti-tTG2 antibodies) is suggested [111,123]. Recovery of the intestinal mucosa is thought to have happened as a result of appropriate adherence to the GFD after laboratory testing results are situated in the normal range, which can occasionally take two years or longer [77,115]. It is important to note that the sensitivity of celiac serology is inadequate to identify minor or sporadic dietary errors [119]. A lot of specialists use follow-up serologies for monitoring diet compliance, and regretfully, results unambiguously demonstrate that serology at follow-up exhibits a weak connection with mucosal healing; as a result, depending exclusively on serology may understate CD activity [120].

Periodic testing for anti-tTG2 IgA and anti-DGP IgA, together with other laboratory tests (such as minerals and vitamins assessments) and symptom evaluation, is the recommended way to track therapy compliance, according to the World Gastroenterology Organization Global Guidelines [124], with The Indian Council of Medical Research also suggesting serologic testing, as it may provide important information after six months and a year following the diet’s introduction [125]. According to the guidelines published by NICE, it is not sufficient to rely just on serological testing to ascertain if an individual has cut gluten out of their diet, and more factors should be reviewed (such as bone mineral density, compliance and adherence to the GFD, symptoms, the possibility of chronic issues and concomitant conditions, etc.) [126].

Numerous surrogate indicators are available to gauge GFD compliance, such as clinical symptom assessment, patient self-report of adherence, food history, dietitian review, or serologic screening tests [75]. Most current recommendations suggest performing serological tests to verify and assess GFD adherence, with these tests also acting as surrogate markers of mucosal recovery in children and adults [77]. Although they are easy to use, minimally invasive, patient-preferred, very specific, and sensitive, these surrogate indicators have a low predictive value for intestinal healing [75,119]. Unfortunately, celiac antibody tests show little sensitivity in identifying chronic mucosal lesions in patients on a GFD despite being routinely used for regular monitoring of CD patients. Furthermore, these procedures were never authorized for this use [127]. Some use a dietary questionnaire or interview in order to address adherence to therapy, but the major downside of this method is the subjectiveness of the information provided by the patient [75,119,128].

Children who have this condition are at risk for malnutrition, but with careful observation, it is possible to avoid needless problems and complications [111]. It was reported that children with CD frequently struggle to follow the GFD and that roughly one-third of patients think the GFD tastes either poor or acceptable, with a quarter of the young people agreeing. Dislike of the way the GFD tastes may be a significant barrier to rigorous GFD adherence, particularly in minors [32]. In their study, Mouslih et al. observed that age has a substantial correlation with compliance, with teenagers being the least cooperative [129]. Parents’ attitudes and comprehension of the condition, its potential repercussions, and the benefits of strictly adhering to the GFD demonstrated improved adherence to the diet among minors [32]. According to Kostecka et al., individuals who had a broad spectrum of CD symptoms, mostly digestive in nature, tended to follow a GFD more rigorously, and their rate of adherence was high [57]. According to the American College of Gastroenterology Guidelines (ACG), within two years of beginning a GFD, 95% of children show signs of intestinal recovery. Due to their greater rates of mucosal healing on a GFD and issues with performing endoscopy in that age group that change the procedure risk-benefit ratio, the decision to do a follow-up biopsy for these patients may be variable. As of right now, a follow-up biopsy after a GFD is not advised for children who are asymptomatic, and monitoring should be done by using the other available methods [114].

In the digestive tract, gluten transforms into GIPs, that are known to cause CD. They are resistant to intestinal digestion and can interact with the CD patient’s immune system to cause an autoimmune reaction [75,113,130]. In the past, no reliable test has been shown to be helpful in objectively identifying gluten consumption [131]. However, as of recently, a relatively new method of monitoring GFD compliance has been developed, and it refers to the direct detection of GIPs (focusing on 33-mer-equivalent gliadin peptide epitopes) found in urine and feces after gluten consumption. This detection is possible by using monoclonal antibodies, being an objective, highly sensitive, quantitative, and non-invasive method to assess dietary adherence [17,40,75,115,127]. The test has a detection limit of as little as 50 mg of gluten ingested, making it highly accurate [128]. Unfortunately, a connection between villous atrophy and positive GIPs tests has not been demonstrated, therefore intestinal damage cannot be addressed with this marker [119]. It is important to note that even among patients who declare strict adherence to the GFD, undetected dietary mistakes are widespread, and research has shown that 70% of patients who tested positive for GIPs also tested negative for anti-tTG2 IgA antibodies, indicating that these dietary errors may go undetected if anti-tTG2 IgA is the only implemented test [120,132].

It is frequently challenging to assess GFD compliance, and most often, prolonged gluten exposure occurs inadvertently. Regardless of how cautious a patient is, exposure can still happen via cross-contamination or simply because of ignorance concerning one’s diet. Straightforward immunological tests based on GIPs analysis from human samples might be used to address this and several other difficulties, such as identifying unintentional deviations from the GFD and patient compliance before any anatomic damage has been done. GIPs analysis can demonstrate gluten consumption or contamination even during the diagnosis of CD and after following a GFD for an extended time; it can be used for both symptomatic and asymptomatic patients, as well as for NCGS individuals [75,120,130,131]. Furthermore, it might be utilized to confirm gluten ingestion during a gluten challenge and to prevent CD from being misdiagnosed [120]. In order to identify dietary violations, GIPs testing is more effective than dietary questionnaires, serology tests, and clinical manifestations [127,131]. The implementation of GIPs as a GFD adherence evaluation method might facilitate the determination of dietary compliance and lessen the requirement for further intrusive follow-up methods since GIPs are only released in feces and urine after consuming gluten [120,127,130].

### 2.7. Reintroduction of Gluten—Is It Possible?

Though this is not wholly supported by research and specialists in the field, it is believed that certain individuals can develop tolerance to gluten over time [90]. Studies have shown the presence of a “latent” variant of CD, where the histology normalizes, as well as the serology, and symptoms disappear over time without reappearing when eating a normal GC diet. People with asymptomatic CD are still advised to adhere to a GFD, with the understanding that some may develop gluten tolerance that requires ongoing evaluation since it may not last forever [17].

A lifetime GFD is, for now, the only available therapeutic option for CD, and all experts suggest following it. Interestingly, there have been a few isolated accounts of afflicted people experiencing a full recovery from their illness while following a GFD for an extended time and returning to a regular diet. Bakhshipour et Rafaiee demonstrated that some adults with CD can regain a normal mucosa (after almost five years of GFD) without experiencing a relapse of any clinical or biological indications of CD following a prolonged time of consuming a GC diet again. They have presented 5 cases of both men and women whose diseases have been remitted. It is crucial to understand that since the condition is autoimmune, the remission state may not result in complete healing and that clinical manifestations may resurface over time [133]. Gluten reintroduction testing should only be considered in individuals with asymptomatic CD and normal intestinal histology; in patients with symptoms, a GFD is required. However, more research is needed to elucidate the safety of reintroducing gluten into such patients’ diets [17].

## 3. Non-Celiac Gluten Sensitivity

### 3.1. Definition, Diagnosis, Symptoms, and General Presentation

Functional, chronic, non-specific, non-allergic gastrointestinal symptoms are poorly understood, and recently, a variety of diagnoses with continually changing labels, such as IBS-like diseases, have been put forward. The most often reported multi-symptom illnesses are small intestine bacterial overgrowth, functional dyspepsia, and IBS. These disorders have a vague clinical overlap [116]. Due to the lack of objective diagnostic criteria, a new clinical syndrome known as “non-celiac gluten sensitivity” has been reported in the range of GRDs. Its incidence varies greatly, ranging from 0.6% to 6% [75,94,116,134]. The primary constraint on NCGS prevalence statistics is the dearth of trustworthy diagnostic biomarkers. Some specialists believe that between 0.5% and 15% of people have NCGS, and that it is highest in the third and fourth decades of life [135]. It is characterized by the induction of symptoms and morphological characteristics upon gluten oral intake, even in the absence of WA and CD. Since each condition has various requirements for adhering to the GFD, both differential diagnosis and patient monitoring have become more challenging [75]. Approximately 13% of NCGS patients are first-degree relatives of CD patients, and it is more frequent in women than in men (W:M = 2.5:1) [1,134,136]. It seems that neurological illnesses, autoimmunity, eating disorders, and functional gastrointestinal diseases are other risk factors for this condition [137]. It also appears that NCGS is a complex condition involving both hereditary and environmental factors, such as ingesting grain proteins [138].

In the absence of CD or WA (i.e., without CD-specific serology, villous atrophy or increased IgE serum levels), patients with NCGS typically describe a broad spectrum of intestinal and extraintestinal symptoms that appear soon after consuming GC foodstuffs [134,139,140]. There is currently a consensus that a non-autoimmune, non-allergic process exists despite the fact that the pathogenetic processes behind the origins of NCGS remain insufficiently comprehended [139]. The patients experience symptoms, which either fade away after following a GFD or become better when gluten is removed from the diet (but return when gluten is reintroduced) [139,141].

Several signs and symptoms can be present, such as diarrhea/constipation, pyrosis, nausea, gut inflammation, vomiting, aerophagia, gastroesophageal reflux disease, flatulence, bloating, abnormal intestinal peristalsis, abdominal discomfort and distension. Fatigue, weight loss, joint/muscle discomfort or pain, stomatitis, migraines, neuroinflammation, anxiety, ataxia, depression, anemia, weight loss, skin rashes, eczema, dermatitis, headaches, limb numbness, and foggy thinking are some of the extraintestinal symptoms, but the intestinal ones are more predominant [136,139,140,141,142]. Neurological symptoms found in NCGS are believed to emerge from disruptions in the gut–brain axis, which is critical. Comprehension concerning the signs, symptoms, and underlying causes of NCGS requires a better understanding of the interaction between the GI tract and the brain [141]. After removing gluten from the diet, all of these symptoms may go away on their own, and they may all flare up a few hours to a few days after consuming gluten again: gastrointestinal symptoms predominating over the extraintestinal ones in this situation as well [113,134,135,139].

Although NCGS incidence appears to have increased over time, correct diagnosis has been challenging due to a lack of diagnostic biomarkers and a lack of knowledge about the disease. A lot of NCGS patients begin a GFD without first seeing a doctor. NCGS is still controversial since the diagnosis is complicated by symptom overlap with other disorders, mainly IBS and CD [90,113]. Therefore, these similar conditions, including WA, must be initially ruled out by serological and histological evidence in order to establish NCGS as a diagnosis. However, similar conditions cannot always be sufficiently differentiated; therefore, it was observed that after reevaluating patients following a gluten challenge or after conducting more in-depth diagnostic testing, up to 20% of NCGS individuals were ultimately reclassified as CD patients [143]. In contrast to CD or WA, NCGS has an enigmatic etiology. It is possible that eating gluten triggers an innate rather than adaptative immunological response [113,144], in comparison with CD, where gluten triggers both [141]. However, no genetic predisposition or specific antibody production has been discovered; therefore, NCGS is a diagnosis based only on the patient’s clinical reaction to gluten in the absence of specific serologic and histologic evidence [77,141]. When all other possible explanations for the symptoms have been exhausted, NCGS is primarily a diagnosis of exclusion [90] or just a clinical diagnosis since there are no particular laboratory tests to be conducted, in order to identify it [145].

Higher levels of anti-tTG2 IgA antibodies do not correlate with NCGS but are associated with CD, and it is recommended that patients do a follow-up gluten challenge, ideally in conjunction with a placebo challenge in order to get the NCGS diagnosis [3]. Currently, a crossover, double-blind, placebo-controlled gluten challenge serves as the gold standard diagnostic test since there are no specific biomarkers available [137,139]. This test could be conducted in a clinical environment (yet it is not practicable to conduct it in the present clinical settings, but only in clinical trials) [6,77], whereby experts give the patient 8 g of gluten (roughly two slices of bread), or a placebo, which is the identical food product without gluten, for one week at a time, separated by one week when the patient is on a GFD. Over the course of three weeks, symptoms are documented [77,97]. It is currently unknown precisely how this medical condition should be addressed, and the pathophysiological mechanisms are not well understood, in addition to a lack of biomarkers and a definitive pathologic diagnosis [30,144].

It is well known that GC foods cause unfavorable reactions in patients with NCGS. However, unlike in the case of CD, which is characterized by villous atrophy and cryptic hyperplasia, these reactions do not result in an autoimmune enteropathy [139,144]. In 1978, Ellis and Linaker documented for the first time gluten sensitivity in the scientific literature [143], and it was also mentioned later in 1980 by Cooper et al. [134,139,144]. After that, it gained more attention due to being more precisely defined and having demonstrated a pathophysiology distinct from CD (NCGS having an innate immune response rather than adaptive) [1,134,144] before almost vanishing from literary works for a number of decades, until 2010 [144].

It is crucial to differentiate between wheat sensitivity and gluten sensitivity since wheat contains other additional components, such as amylase trypsin inhibitors (ATI) [6,30,136,138] (natural pesticides or “pest-resistant molecules” that trigger the innate immune reaction), that may be the cause of the symptoms [15,30]. Fructans found in wheat belong to a class of foods called fermentable oligosaccharides, disaccharides, monosaccharides, and polyols, or FODMAPs for short, which are recognized to cause gastrointestinal discomfort, since they are absorbed slowly and easily fermented by intestinal bacteria, in people with IBS and possibly in those with NCGS [134,136,146]. It has been demonstrated that a low-FODMAP diet is an effective treatment for reducing the functional gastrointestinal symptoms associated with IBS and NCGS. For those who self-report NCGS, this diet seems to be more beneficial than the GFD [146]. A difficulty with nomenclature arises from the increasing evidence that additional proteins specific to GC cereals can initiate an innate immune response and cause NCGS [134,147]. Since it is unclear if gluten is the primary source of the symptoms, it has been suggested by some researchers that the illness should be referred to as non-celiac wheat sensitivity (NCWS) rather than NCGS [144,148], but in this article, it is referred to as NCGS.

### 3.2. Pathophysiology

Currently, there is a lack of data on the likelihood that gluten consumption contributes to gastrointestinal symptoms in individuals who do not have CD. Patients exposed to a GC diet are known to have a marked exacerbation of their overall symptoms, exhaustion, and stomach discomfort; however, a clear reason for this has not been found. Additionally, the impact of gluten was investigated in individuals with IBS whose primary symptom was diarrhea. The results showed that consuming gluten increased the frequency of stools, with the effect being stronger in patients who had the HLA-DQ2 or HLA-DQ8 genotype. However, the mechanism by which gluten impact may differ from that of CD and induce digestive issues in individuals with NCGS is confusing [15,134]. HLA haploid genotypes are present in approximately 50% of NCGS individuals [143]. As of now, there is no clear proof of altered adaptive immunity or increased intestinal permeability [134], with some researchers claiming that the intestinal permeability is normal [30] and others disagreeing and stating that it is increased [143]. However, this condition was linked to intestinal inflammation, higher levels of interferon-gamma (IFN-γ), and intraepithelial lymphocyte numbers, which indicate enhanced immunological activity in the intestinal mucosa, stipulating that adaptive immunity may actually have an unfavorable function in this condition [134]. It is also suggested that adaptive immunity may have a role in the development of NCGS due to the discovery of IgG anti-gliadin antibodies (AGA) in approximately 50% of NCGS patients [134,136,141,142]. IgA-AGA are infrequently found [143]; however, the potential involvement of toll-like receptors (e.g., TLR-1 and 2) in NCGS pathophysiology supports the innate immunity theory [113,135]. While these findings are important and helpful in trying to understand the pathophysiology of NCGS, they make it even more confusing yet intriguing.

While some researchers maintain the idea that NCGS is not a distinct clinical entity and instead consider it a subset of IBS (since 1/3 of IBS patients have gluten sensitivity) [137,144], others have shown that FODMAPs are the source of gastrointestinal symptoms [144]. Most often, NCGS is acknowledged as a separate clinical condition from IBS that has similar manifestations with both IBS and CD but without the intestinal damage characteristic of CD [137]. Furthermore, since there is a distinction between food sensitivity and food intolerance, it is critical to define those terms precisely. When comparing the definitions of food intolerance (gastrointestinal symptoms secondary to the colonic microbiota’s fermentation process of carbohydrates due to its incapacity to properly metabolize certain nutrients) and food sensitivity (an immune response to antigens derived from nutrients that cause gastrointestinal and extraintestinal symptoms), it should be unambiguous that IBS and NCGS are separate diseases with some overlapping features that can make diagnosis difficult [144].

### 3.3. Nutritional Deficits Associated with NCGS

According to Silvester et al., nutrient deficiencies are found in only 18.4% of NCGS individuals, such imbalances being more common among CD patients [3]. According to Carroccio et al. and their research results, osteopenia or osteoporosis was present in almost 50% of NCGS individuals. It was also shown that individuals with this condition who also had other food sensitivities had a higher incidence of bone density loss, which was linked to a low BMI. Furthermore, a reduced daily consumption of dietary calcium was noted in people with NCGS [148]. Moreover, Volta et al. showed that inadequate amounts of ferritin, folic acid, and vitamin D were found in individuals with potential NCGS, according to their laboratory studies [136]. Since being on a GFD without professional healthcare oversight risks low fiber intake, micronutrient and vitamin insufficiencies, and macronutrient imbalances—nutritional guidance is crucial [138]. Since NCGS patients are more likely to experience osteoporosis, anemia, and weight loss, it is possible that some of them have an early form of CD that will manifest much later. Furthermore, people with NCGS have a greater incidence of autoimmune diseases and atopic illnesses than those with IBS [6].

### 3.4. Therapy and Follow-Up

The GFD is recommended as the main successful treatment for the management of NCGS, together with the advice and assistance of a trained dietician [137,138]. The most recent therapeutic strategy for NGCS involves avoiding gluten for the rest of one’s life [137]. Follow-up strategies are not clear and well implemented, but since IgG-AGA levels dissipate along with symptom relief in a matter of six months on a GFD, it has been proposed that these antibodies might serve as a potential indicator of compliance in NCGS patients since the ongoing presence of the AGA signifies a low adherence to diet therapy and gluten consumption [140].

It has been hypothesized that NCGS could be temporary; therefore, it is advised to stick to the GFD for 1–2 years before retesting for gluten sensitivity [77,135]: a procedure where patients might undergo a gluten challenge, where they can try to reintroduce modest and gradually increasing quantities of gluten into their diet. However, the precise amount that each patient is able to tolerate must be determined on an individual basis by keeping track of the quantity of gluten that they consume and any relevant extraintestinal or gastrointestinal symptoms that may arise [135,149]. Depending on how severe their symptoms are, NCGS patients may choose to continue the GFD indefinitely, whereas CD sufferers are required to maintain a strict GFD for the rest of their lifespans [77]. For NCGS, a less rigorous GFD is seen to be the answer (minimal quality of evidence), but for CD, a highly rigid diet is necessary [113]. Furthermore, individuals with NCGS who have experienced symptom remission after switching to a low-FODMAP diet can be advised to reevaluate their reaction to food by adding high-FODMAP items to their diets 4–6 weeks after beginning a low-FODMAP diet. However, because this diet has been linked to a poor intake of micronutrients and naturally occurring antioxidants, it should be carefully evaluated how strictly and for how long it should be followed. Additionally, it has been observed that FODMAPs have a prebiotic impact on intestinal bacteria, promoting the development of *Lactobacilli* and *Bifidobacteria* while inhibiting the colonization of pathogenic bacteria (*Bacteroides* spp., *Escherichia coli*, and *Clostridium* spp.). Thus, for patients following a low-FODMAP diet, nutritional supplementation with prebiotics and vitamins is generally advised [135].

## 4. Intestinal Microbiota Modulations and Dysbiosis

The human body’s biggest concentration of microorganisms, including viruses, bacteria, fungi, archaea, and protozoa are found in the gastrointestinal system, and their structure is constantly changing. The word “microbiome” refers to the complete human microbiota’s genome [150,151]. According to Calabriso et al., “about 90% of the gut bacteria is represented by the phyla *Bacteroidetes* (including genera *Bacteroides* and *Prevotella*) and Firmicutes (encompassing genera *Lactobacillus*, *Bacillus*, *Clostridium*, *Enterococcus*, *Ruminococcus*, *Eubacterium*, *Faecalibacterium*, and *Roseburia*), followed in much lower abundance by *Actinobacteria* (*Bifidobacterium*), and *Verrucomicrobia* (namely *Akkermansia* spp.)” [135], and, to a lesser degree, *Proteobacteria* [152]. In contrast to healthy individuals, patients with CD and NCGS have different gut microbial patterns. It is believed that there are multiple parallels in the bacterial makeup of the gut microbiota between people with CD and those with IBS and fewer similarities between people with NCGS and those with IBS [153].

The old adage that all diseases originate in the gut is sometimes very accurate since dysbiosis, which is a result of modifications to the normal microbial balance in terms of both composition and function, can be the first step toward a variety of disorders [150]. Because the immune system and gut microbiota interact, changes in the microbiota of the gastrointestinal tract have been associated with a number of autoimmune diseases, including CD [154]. The gut microbiome is often altered in CD, showing changes in microbial diversity, composition, and function. In particular, a reduction in microbial diversity, an imbalance in the proportional distribution of specific bacterial taxa, and modifications to microbial metabolic activity are frequently seen in those with CD. As previously mentioned, the pathophysiology of the condition is characterized by alterations that may lead to inflammation, immunological dysregulation, and compromised intestinal barrier function [93,154,155,156].

It has been reported that while beneficial Gram-positive microorganisms like *Clostridia*, *Lactobacilli* and *Bifidobacteria* are less abundant in celiac patients, the presence of CD is also linked to increased levels of numerous Gram-negative bacterial species such *Bacteroides*, *Escherichia*, *Akkermanisia*, *Staphylococcaceae*, *Clostridium*, *Enterobacteriaceae*, *Neisseria*, *Klebsiella*, *Salmonella*, *Schighella* and *Prevotella* [93,150,151,154,157,158]. The prevalence of bacteria with anti-inflammatory characteristics, such as *Faecalibacterium prausnitzii* and *Streptococcus thermophilus*, is much lower among individuals prior to the onset of CD. Additionally, even viral and fungal dysbiosis have been reported in CD, indicating a change in certain taxa [93,150]. For instance, a possible major link between CD and the common yeast species *Candida albicans* has been shown. It has been established that gliadin protein-1, which is expressed by *Candida albicans*, is structurally similar to the gliadin protein present in gluten, which is known to induce CD signs and symptoms in genetically predisposed individuals. Due to their structural similarities, researchers have hypothesized that the type of yeast might be a trigger for the development or aggravation of CD [159]. It was also discovered that the abundance of mucosal samples is higher in taxa such as *Sacchharomycetes* and *Candida* in celiac patients compared to healthy controls [93].

The topic of whether CD causes dysbiosis or if dysbiosis causes this particular medical condition is one that is frequently debated. The development of CD has indeed been linked to dysbiosis of the microbiome. There is disagreement over the precise microbial alterations seen in this disorder despite the data pointing to the importance of gut microbiota for CD etiology. It was discovered that CD patients could have an increase in *Actinobacteria* species in the upper gastrointestinal tract (GIT) and an increase in *Proteobacteria* species in the lower GIT. Additionally, following a GFD improves the dysbiosis associated with CD by reducing some *Betaproteobacteriales* and increasing beneficial gut bacteria [155]. Studies show lower levels of *Coprocococcus* and *Enterorhabdus* in individuals genetically at risk of various autoimmune disorders [158]. Infants who are genetically predisposed to CD show decreased amounts of several *Coprococcus*, *Streptococcus*, *Parabacteroides*, *Veillonella* species, and *Clostridium perfringens*, suggesting that the onset of CD in babies could be linked to gastrointestinal dysbiosis [157]. It has been suggested that the dysbiotic sequela of cesarean delivery is a risk factor for the development of various diseases [160], including CD. This suggestion is further reinforced by the correlation between cesarean section and lower levels of some beneficial species of *Bacteroides* (*Bacteroides vulgatus* and *Bacteroides dorei*) and *Parabacteroides* and higher levels of *Enterococcus faecalis*; that, when compared with vaginal delivery [157].

By regulating the interaction between the host immune system and gluten or environmental stimuli, the gut microbiome appears to play a major role in the emergence of CD. The discovered changes suggest a move from the preclinical phase of the illness to a loss of gluten tolerance and the eventual beginning of CD. Signs and symptoms of CD could, at least in part, be understood as due to ongoing microbiota dysregulation. For instance, we can look at the neurological signs of CD, and the prevailing consensus is that they might happen during the early stages of the disease or as it progresses. One of the primary symptoms of CD is migraines, and it has been suggested that disruptions in the gut–brain axis could be to blame. Also, it has been shown that individuals with a CD diagnosis have a pro-inflammatory intestinal microbiota [151]. The connection between migraine and CD may be explained by the neurological symptoms of the condition, which are thought to be caused by a systemic inflammatory response, being tightly related to the intestinal microbiota abnormalities in individuals with CD [156].

Modulation of the microbiota may be useful as a therapeutic and preventive measure for GRDs [151]. Although the GFD can ameliorate the duodenal pathohistological and clinical manifestations, further treatment is still required since intestinal dysbiosis persists in CD even on a GFD [93]. It is thought that a GFD or a gluten-restricted diet could be one of the main factors leading to dysbiosis [150,161]. In adult individuals with CD, probiotics, prebiotics, and fecal microbial transplants have shown promise in reestablishing a healthy gut microbial balance [93]. Recent studies have yielded preliminary data that suggest that the use of prebiotic and probiotic products, following gluten restriction, may reduce intestinal hyperpermeability, enhance the digestive tract immune response, and restore normal villous architecture. Subsequent investigations into the variety, dosage, and duration of probiotic intake are of crucial importance, and research on the connection between altered gut microbiota and the initial development of CD is well underway [162]. It is still unclear how the gut microbiota affects the immune system’s response to gluten consumption and exactly what role it plays in CD patients. In spite of this, it is recognized that the prevention, onset, and management of CD can all benefit from a healthy gut microbial composition [163].

Gut dysbiosis, for the NCGS population, is one of the major factors that might lead to loose stool, chronic inflammation, intestinal hypersensitivity, and immunological dysfunction. Lower *Bacteroidetes* and *Lactobacillus* species abundance was demonstrated by Naseri et al. in their study. Additionally, they observed a noteworthy rise in the *Firmicutes* to *Bacteroidetes* ratio in both NCGS and CD subjects when compared to healthy controls [164]. According to a Mexican study, the duodenal microbiota of NCGS patients had higher levels of the genus *Actinobacillus* and the family *Ruminococcaceae*, whereas the duodenum of CD patients had higher levels of the genus *Novispirillum*. The results also showed that after the NCGS group followed a GFD for 4 weeks, an unexpected and increasing abundance of duodenal *Pseudomonas* was documented [145]. According to *Leccioli* et al., it is proposed that a specific dysbiotic profile, marked by a reduction in butyrate-producing *Firmicutes* and/or *Bifidobacteria*, is the underlying cause of NCGS, resulting in low intestine butyrate levels, indicating a particular dysbiotic profile. This imbalance impairs the gut’s capacity to regulate inflammatory stimuli originating from the food (such as ATI) and the resident microbiota (like lipopolysaccharides). Because butyrate stimulates the proliferation and differentiation of epithelial cells, assists in intestinal injury repair and mucous secretion, and inhibits microbial translocation, it is critical for preserving intestinal health. Reduced levels may result in increased intestinal permeability and decreased gut integrity [143]. How effectively a person reacts to a GFD may depend on their intestinal dysbiosis since the efficacy of the GFD in treating NCGS symptoms can be increased by reestablishing a healthy gut microbiota via nutrition, biotics, or other therapies. In this population, dysbiosis aggravates symptoms by inducing immunological reactions, raising intestinal permeability, and promoting systemic and neuroinflammation [141].

Interestingly, while a GFD could benefit the overall composition of the intestinal microbiota in both NCGS and CD patients, the precise alterations and their degree vary across these illnesses. Even when following a GFD, CD patients still frequently show dysbiosis, whereas NCGS patients may show distinct modifications in their microbiota that are associated with symptom improvement. Comprehending these distinctions is essential to formulating effective therapeutic approaches that correctly target gut health in various demographics. A GFD promotes the development of pathogenic bacteria, such as *Enterobacteriaceae* and *Escherichia coli*, surpassing the development of beneficial organisms, such as *Bifidobacteria*, in healthy individuals with no GRDs. Given that dysbiosis persists in CD patients on a GFD, dietary intervention alone may not be sufficient to address the issue. It could be possible to restore a more regulated gut microbiota with the use of probiotics, prebiotics, and other microbiota-targeted pharmaceuticals. Especially for individuals who are not responding well to a GFD alone, personalized methods of treating intestinal microbiota disturbances in both CD and NCGS patients may enhance health outcomes and quality of life [152].

### Prebiotics, Probiotics, Synbiotics, Postbiotics

Probiotics are defined by the International Scientific Association of Probiotics and Prebiotics (ISAPP) as living microorganisms that provide a host with health benefits when given in sufficient quantities. The majority of them are represented by *Lactobacillus* and *Bifidobacterium* species [165]. Prebiotics are pictured by ISAPP as a substrate that hosts gut bacteria specifically used to promote human health. Therefore, they support intestinal wellness since they are non-digestible oligosaccharides that stimulate beneficial microorganisms in the large intestine, such as *Lactobacillus* and *Bifidobacterium* species [166]. Moreover, synbiotics are a combination of living microorganisms (probiotics) and substrate(s) (prebiotics) that are specifically used by the host microorganisms to promote the host’s well-being and health [167]. A postbiotic, according to ISAPP, is a “preparation of inanimate microorganisms and/or their components that confers a health benefit on the host”. Postbiotics are microbial cells that have been purposefully inactivated, either with or without metabolites or parts of cells, and they have also been shown to have positive effects on health [168].

Probiotics have been shown to have a broad spectrum of beneficial health benefits, which have been found to be strain-specific. *Bifidobacterium* and *Lactobacillus* are the two most common genera used and studied in probiotic applications [169]. Because of their established ability to enhance good health, additional microorganisms from the genera *Saccharomyces*, *Enterococcus*, *Lactococcus*, and *Propionibacteria* are added to the selection of probiotics [170]. According to a recent study, we are provided with evidence of the production of enzymes through the use of microbial consortia consisting of strains of *Bacillus* and *Lactobacillus*. Under gastrointestinal circumstances, these consortia were reported to efficiently break down gluten into non-immunogenic and non-toxic peptides. According to the study, these microbial consortia and microbial proteolytic enzymes may be able to detoxify GIPs by hydrolyzing them, which would help people with CD and NCGS digest food with less difficulty [171].

For the food industry, the rapidly developing field of pre- and probiotics offers significant development opportunities [170]. They can be added to food products (such as dough-based products, cereal, and drinks), which is a very convenient way of delivery [166]. Probiotic efficiency and commercial feasibility depend on the particular strains or cultures used. Foodstuffs that are naturally high in probiotics (such as fermented products), prebiotics (banana, asparagus, onion, garlic, tomato, peas, beans, etc.), synbiotics (found in some fermented dairy products, for example), or are artificially enriched exert great influence by supporting a microbiota conducive to gut health and even leading to improvements in the lipidic profile of the host and reduction in inflammation [170,172,173].

Synbiotics, which have both probiotic and prebiotic qualities, were developed to help the probiotics survive in the gastrointestinal system in the event of certain potential challenges. The idea behind synbiotics is to increase the probiotic bacteria survival rates and activity in the gut by giving them the prebiotics they need to flourish. Consequently, when both ingredients are combined properly in a single product, the result should be better than their individual effect [173]. On the other hand, numerous postbiotics currently in use are inactive strains of well-established probiotic taxa found in certain genera of the genus *Bifidobacterium* or the family *Lactobacillaceae*. Moreover, for the inactivated form of a microbial strain or consortia to be recognized as a postbiotic, it does not have to satisfy the requirements for being a probiotic while alive. *Apilactobacillus kunkeei*, *Saccharomyces boulardii*, *Eubacterium hallii*, *Bacteroides xylanisolvens*, *Bacteroides muciniphila*, *Faecalibacterium prausnitzii*, *Clostridial cluster* IV and XIVa, and *Akkermansia muciniphila* strains have all been studied for possible positive impacts while in an inanimate form [168]. Moreover, prebiotics have been proven in recent studies to have beneficial properties that may enhance the gut epithelium barrier function and lessen the pro-inflammatory profile in CD individuals [174].

The complex interactions between the gut microbiota and various probiotics, prebiotics, synbiotics, and postbiotics, as well as their subsequent potential benefit for individuals with GRDs, have been the subject of much research in later years. The use of these biotics might provide a viable and very promising dietary adjuvant to the GFD as a novel therapeutic approach to better manage GRDs and the associated dysbiosis. Moreover, analyzing the patient’s microbiome might become a new step in the diagnostic journey. The above findings may benefit all patients suffering from GRDs, and more research is needed to better understand their implications.

## 5. Conclusions

Gluten-related illnesses impact many individuals; they are complex and represent a multifaced and extensive concern. The intricate connections between gluten and the body’s immune system have been examined in this narrative review, with a special emphasis on two diseases: CD and NCGS. It is important to understand and differentiate these disorders, their manifestations, and particularities to facilitate early diagnosis, and this paper supports this direction.

The GFD continues to be the gold-standard therapy, playing a crucial role in the management of these disorders, although it is clear that, for some patients, it may not suffice as a standalone intervention. Recent research shows that incorporating biotics—probiotics, prebiotics, synbiotics, and postbiotics—into meal plans may help regulate gut microbiota and enhance patient outcomes [152].

In order to create more appealing and accessible GF goods, as well as to expand our understanding of how biotics might be successfully included in treatment programs for GRD patients, more research is needed. Furthermore, a promising path toward more individualized and efficient GRD care is provided by the potential of microbiome investigation as a diagnostic instrument. All things considered, even if a lot of progress has been made, further research and development into diagnosis strategies, dietary plans, and therapy approaches are necessary to improve the quality of life for those with GRDs.

## Figures and Tables

**Table 1:** Protein-dense sources as ingredients for GF foodstuffs production.

Source	Flour Protein (g%)	Reference
Potato	94.06	[50]
Pea	80.19	[50]
Faba bean	61.25	[51]
Soy	56.60	[52]
Carob	55.04	[50]
Lentil	28.10	[53]
Chickpeas	23.37	[54]
Buckwheat	22.52	[51]
Amaranth	19.40	[55]
Quinoa	19.00	[55]
Teff	13.30	[56]
Sorghum	13.08	[47]
Millet	11.90	[55]
Rice	7.90	[55]
Maize	7.45	[54]

**Table 2:** Histopathologic Marsh-Oberhuber classification.

Grade	Villous Atrophy	Crypt Hyperplasia	Intraepithelial Lymphocytic Infiltration
0	−	−	−
1	−	−	+
2	−	+	+
3a	+ (mild)	+	+
3b	+ (moderate)	+	+
3c	+ (severe)	+	+
4	+ (severe)	+	−

“−” Absent; “+” Present.
